# Clinical Insight Into Latent Variables of Psychiatric Questionnaires for Mood Symptom Self-Assessment

**DOI:** 10.2196/mental.6917

**Published:** 2017-05-25

**Authors:** Athanasios Tsanas, Kate Saunders, Amy Bilderbeck, Niclas Palmius, Guy Goodwin, Maarten De Vos

**Affiliations:** ^1^ Usher Institute of Population Health Sciences and Informatics Medical School University of Edinburgh Edinburgh United Kingdom; ^2^ Institute of Biomedical Engineering Department of Engineering Science University of Oxford Oxford United Kingdom; ^3^ Department of Psychiatry University of Oxford Oxford United Kingdom; ^4^ Oxford Health NHS Foundation Trust Oxford United Kingdom

**Keywords:** bipolar disorder, borderline personality disorder, depression, mania, latent variable structure, mood monitoring, patient reported outcome measures, mhealth, mobile app

## Abstract

**Background:**

We recently described a new questionnaire to monitor mood called mood zoom (MZ). MZ comprises 6 items assessing mood symptoms on a 7-point Likert scale; we had previously used standard principal component analysis (PCA) to tentatively understand its properties, but the presence of multiple nonzero loadings obstructed the interpretation of its latent variables.

**Objective:**

The aim of this study was to rigorously investigate the internal properties and latent variables of MZ using an algorithmic approach which may lead to more interpretable results than PCA. Additionally, we explored three other widely used psychiatric questionnaires to investigate latent variable structure similarities with MZ: (1) Altman self-rating mania scale (ASRM), assessing mania; (2) quick inventory of depressive symptomatology (QIDS) self-report, assessing depression; and (3) generalized anxiety disorder (7-item) (GAD-7), assessing anxiety.

**Methods:**

We elicited responses from 131 participants: 48 bipolar disorder (BD), 32 borderline personality disorder (BPD), and 51 healthy controls (HC), collected longitudinally (median [interquartile range, IQR]: 363 [276] days). Participants were requested to complete ASRM, QIDS, and GAD-7 weekly (all 3 questionnaires were completed on the Web) and MZ daily (using a custom-based smartphone app). We applied sparse PCA (SPCA) to determine the latent variables for the four questionnaires, where a small subset of the original items contributes toward each latent variable.

**Results:**

We found that MZ had great consistency across the three cohorts studied. Three main principal components were derived using SPCA, which can be tentatively interpreted as (1) anxiety and sadness, (2) positive affect, and (3) irritability. The MZ principal component comprising anxiety and sadness explains most of the variance in BD and BPD, whereas the positive affect of MZ explains most of the variance in HC. The latent variables in ASRM were identical for the patient groups but different for HC; nevertheless, the latent variables shared common items across both the patient group and HC. On the contrary, QIDS had overall very different principal components across groups; sleep was a key element in HC and BD but was absent in BPD. In GAD-7, nervousness was the principal component explaining most of the variance in BD and HC.

**Conclusions:**

This study has important implications for understanding self-reported mood. MZ has a consistent, intuitively interpretable latent variable structure and hence may be a good instrument for generic mood assessment. Irritability appears to be the key distinguishing latent variable between BD and BPD and might be useful for differential diagnosis. Anxiety and sadness are closely interlinked, a finding that might inform treatment effects to jointly address these covarying symptoms. Anxiety and nervousness appear to be amongst the cardinal latent variable symptoms in BD and merit close attention in clinical practice.

## Introduction

Regular monitoring of symptom severity and disease progression in mental disorders is widely encouraged in treatment guidelines [1,2]. This had been typically achieved using patient reported outcome measures (PROMs), that is, self-assessment of mood on standardized questionnaires. Originally, questionnaires were paper-based and more recently computer-based [3,4]; however, recent technological developments have generated considerable interest in capitalizing the wide availability of smartphones to embed questionnaires in purpose-built apps [5-9]. This approach has advantages because mood self-assessment is reported in real time alleviating the issue of recall bias [10].

One approach toward PROMs is to develop generic instruments capturing universal outcomes that are relevant across a wide range of diseases and conditions such as pain and fatigue. This motivated the development of the patient reported outcomes measurement information system (PROMIS), an instrument for self-reporting physical, mental, and social health aspects in the general population [11-13]. Some associated toolbox measures have been developed using the item banks within PROMIS to cover specific populations, for example, those diagnosed with a neurological condition or disorder [14]. Universal measures such as PROMIS are undoubtedly useful for large-scale studies facilitating direct comparisons across diverse cohorts and diseases; however, by design, they are not necessarily sensitive to capturing all the intricate symptom changes of specific diseases. The alternative approach to generic instruments is to develop tailored disease-specific (also known as disease-attributed) instruments that may be of particular significance from a clinician’s perspective for effective assessment and monitoring of symptoms within a specific disease or condition. Both universal PROMs and disease-specific PROMs have merits and shortcomings, and the decision to use either approach depends upon the aims of a study.

In this study, we focus on mining PROMs using disease-specific clinical scales to better understand the underlying symptoms in bipolar disorder (BD) and borderline personality disorder (BPD), comparing findings against healthy controls (HC). BD is characterized by recurrent alternating periods of elated mood (known as mania or hypomania, depending on symptom severity) and depression, which is usually more common [15]. Symptom-free periods in BD are known as euthymia. Symptom management is typically achieved using long-term medication [16], including mood stabilizers and antipsychotics [15]. BPD is characterized by splitting (failing to form a cohesive whole taking into account positive and negative traits for self and others), impulsivity, irritability, negative criticism, difficulty to regulate emotions, depression, anxiety, and anger [17]. The dominant treatment modality is psychotherapy although pharmacotherapy is common in clinical practice. BD and BPD can be clearly distinguished using laboratory measures of social cooperation and reward learning [18] but in clinical practice their distinction can be far more challenging because of the overlap in the diagnostic criteria. Correct diagnosis is critical given the divergent treatment approaches. Mood monitoring is commonly used in both clinical groups although the interpretation of their mood scores has often been challenged as positive responses are thought to reflect very different underlying psychological processes.

A critical aspect of understanding PROMs is deciphering the underlying structure inherent in the questionnaires eliciting the participants’ responses. That is, identifying some characteristics (latent variables) which are not directly observed through the items in the questionnaires but which are inferred through algorithmic processing of the observed items. One of the main advantages of using latent variables is explaining most of the data using a few variables which may be tentatively interpretable. They comprise items grouped together, thus indicating which different symptoms may be related. Hence, latent variables might offer additional insight into the underlying mood symptoms, and suggest new directions for clinical assessment and care.

The aims of this study were to: (1) explore the latent variable structure of a recently introduced psychiatric questionnaire known as Mood Zoom (MZ) [9] to understand better its properties and internal structure, (2) identify differences in the latent variables of the MZ questionnaire for the three studied cohorts (BD, BPD, and HC) and observe how well they differentiate the patient cohorts and benchmark findings against HC, and (3) explore three other widely used psychiatric questionnaires and identify their internal consistency across cohorts and their potential similarities with MZ.

## Methods

### Data

The data were collected as part of a large ongoing research project known as automated monitoring of symptom severity (AMoSS) [9]. We record mood, activity, and physiological variables using a variety of sensors [19,20]. The study is observational and independent of participants’ clinical care: we recruited 141 participants, and their demographic details are summarized in Table 1. The participants were recruited for an initial 3-month study period, with an option to remain in the study for 12 months or longer. The patient cohorts were mainly recruited from other ongoing studies in Oxfordshire or from individuals who had previously registered interest to be involved in future research; in particular, some of the BD participants have had multiple years of experience in mood self-reporting. The age-matched HC were recruited by means of advertising in commonly used forums locally.

We excluded data from participants who either withdrew consent (1 participant) or completed participation without providing at least two months of useful data for all questionnaires (9 participants). We processed data from 131 participants, 120 of whom had provided data for at least three months, and 108 of whom had provided data for at least 12 months. All participants gave written informed consent to participate in the study. All patient participants were screened by an experienced psychiatrist (KEAS) using the structured clinical interview for diagnostic and statistical manual of mental disorders, 4^th^edition (DSM IV) and the borderline items of the international personality disorder examination (IPDE) [21]. The study was approved by the NRES Committee East of England- Norfolk (13/EE/0288) and the research and development department of Oxford Health NHS Foundation Trust.

### Questionnaires for Mood Self-Monitoring

The participants reported their mood on a weekly basis using three validated questionnaires: (1) Altman self-rating mania scale (ASRM) [22] to assess mania, (2) quick inventory of depressive symptomatology (QIDS) self-report [23] to assess depression, and (3) generalized anxiety disorder (7-item) (GAD-7) [24] to assess anxiety. These three questionnaires were completed on the Web using the true colors (TC) system: the participants had been previously registered on the website and would need to provide their log in credentials to securely connect to their TC page. In all cases, the participants were requested to complete ASRM, QIDS, and GAD-7 reporting the average symptoms during the preceding week. The MZ questionnaire [9] was completed on a daily basis using a custom-based smartphone app developed for the needs of the AMoSS project.

ASRM is comprised of 5 items: (1) mood, (2) self-confidence, (3) sleep disturbance, (4) speech, and (5) activity. Items are scored on a 0 (symptom-free) to 4 (present nearly all the time) scale, and the total ASRM is computed by adding up the items in the 5 sections giving rise to the range 0 to 20. Miller et al [25] proposed a cut-off score of 5.5 assess a manic episode.

QIDS is comprised of 16 items, where each item is scored on a 0 (symptom-free) to 3 scale. The items map onto 9 DSM-IV symptom criteria domains for depression: (1) sad mood, (2) concentration, (3) self-criticism, (4) suicidal ideation, (5) loss of interest, (6) energy or fatigue, (7) sleep disturbance, (8) changes in appetite or weight, and (9) psychomotor agitation or retardation. Each domain is either the highest score of a subset of the 16 QIDS items or one of the original QIDS items; see Rush et al for details [23]. Each domain contributes 0-3 points, and adding up these domains gives rise to the QIDS total score ranging from 0 to 27. The suggested clinical ranges are 5 or less denoting normal, 6-10 denoting mild depression, 11-15 denoting moderate depression, 16-20 denoting severe depression, and 21-27 denoting very severe depression [23,26].

GAD-7 is comprised of 7 items which are scored on a 0 (symptom-free) to 3 (nearly every day) scale, with total scores ranging from 0 to 21. Kroenke et al [27] endorsed using the threshold cut-offs at 5, 10, and 15 to denote mild, moderate, and severe anxiety, respectively.

MZ is comprised of 6 items: (1) anxious, (2) elated, (3) sad, (4) angry, (5) irritable, and (6) energetic. Each item is scored on a Likert scale ranging from 1 (“not at all”) to 7 (“very much”). Participants were prompted to complete MZ during the study daily in the evening at a prespecified chosen time.

### Samples Used for the Four Questionnaires

We constructed 4 data matrices to contain the data for subsequent processing, one data matrix for each of the questionnaires. Subsequently, we worked independently on each of those 4 matrices to determine properties applicable to each of the questionnaires.

For ASRM we used a 5719×5 data matrix. There were 2363 samples for BD, 1298 samples for BPD, and 2058 samples for HC.

For QIDS we used a 4871×9 data matrix. There were 2054 samples for BD, 1099 samples for BPD, and 1718 samples for HC.

For GAD-7 we used a 5652×7 data matrix. There were 2208 samples for BD, 1389 samples for BPD, and 2055 samples for HC.

For MZ we used a 44725×6 data matrix (44725 samples and 6 items). There were 17317 samples for BD, 11120 samples for BPD, and 16288 samples for HC.

Any missing entries (~20% as we reported in our previous study [9]) had been removed before reporting these figures.

**Table 1:** Summary of the key demographics of participants in automated monitoring of symptom severity (AMoSS).

	Bipolar disorder	Borderline personality disorder	Healthy controls
Originally recruited	53	34	54
Processed data from	48	32	51
Days in study, median (IQR^a^range)	365 (325; 69-867)	364 (194; 81-858)	363 (191; 80-651)
Age (years), median (IQR range)	38 (19; 18-64)	34 (14 21-56)	37 (20; 19-63)
Gender (male)	17	2	18
Unemployed	7	15	6
Any psychotropic medication	47	23	0
Lithium	19	0	0
Anticonvulsant	19	1	0
Antipsychotic	33	6	0
Antidepressants	17	23	0
Hypnotics	3	2	0

^a^IQR: interquartile range.

### Data Preprocessing

Before processing the data, we standardized entries to reflect individual reporting bias so that they are directly comparable across participants. This preprocessing step was deemed necessary because the same level of mood may be assigned a different item score by different participants, and hence the raw item scores are not directly comparable across participants. Therefore, for each questionnaire, we subtracted from each item entry the mean value of that item per participant. Effectively, this transformed the discrete data matrices into continuous data matrices. This step is particularly useful in combination with the latent variable structure approach described below.

### Extracting Latent Variable Questionnaire Structure Using Sparse Principal Component Analysis

Given a data matrix **X** *N* × *M* that is, a collection of the questionnaire entries comprising *N* samples (observations) and *M* variables (for this study *M* is the number of items of the investigated questionnaire), we wanted to obtain its internal structure, which is potentially governed by some unseen variables. That is, we wanted to project the information inherent in the original items in such a way that we could identify a robust set of some new variables that might offer new or alternative insights into the hidden structure in the data, that is, identify the latent variables.

The mathematical approaches to achieve this can be generally divided into linear and nonlinear methods, depending on how the original variables in the data matrix are combined to derive the latent variables. Although sophisticated nonlinear methods may work well in complicated toy problems, they are often more difficult to interpret than some standard linear projection techniques (which in many practical settings may also work very well). One of the most widely used methods for detecting the latent variable structure of a data matrix is principal component analysis (PCA) [28]. PCA computes linear combinations of the *M* variables, known as principal components. The principal components are projected in orthogonal directions (hence, they are linearly uncorrelated) and successively explain the largest possible remaining variance in the data. The coefficients each variable in **X** contributes toward predicting the principal components are known as the loadings. The PCA structure looks like the following:

P_1_= *l*_11_⋅x_1_+ *l*_12_⋅x_2_+ *l*_13_⋅x_3_+ … + *l*_1M_⋅x_M_

P_2_= *l*_21_⋅x_1_+ *l*_22_⋅x_2_+ *l*_23_⋅x_3_+ … + *l*_2M_⋅x_M_

…

P_M_= *l*_M1_⋅x_1_+ *l*_M2_⋅x_2_+ *l*_M3_⋅x_3_+ … + *l*_MM_⋅x_M_

In the equation, P_1_… P_M_ are the principal components, x_1_… x_M_ are the items in each questionnaire, and *l*_ij_ refers to the loading of the *j* th item contributing toward the computation of the *i* th principal component (and all the *l*_ij_ entries form the loading matrix **L**). Usually, we only want to work on the first few principal components, which explain most of the variance in the data.

In practice, each principal component is a linear combination of all the original variables; that is, the loadings are generally non-zero, and therefore the interpretation of the resulting principal components may be challenging. Ideally the structure (ie, collectively the loadings) should be simple, comprising a few non-zero entries associating a small subset of the variables in subset of the **X** with the principal components, and still maximizing as much of the explained variance in the data as possible. Hence, researchers have developed various sparse PCA (SPCA) approaches to promote principal components that are dependent only on a small set of variables in the original data matrix. Inherently, there is a compromise to be made between the interpretability of the principal components and the explained variance [29-32].

In this study, we followed the methodology proposed in Hein and Buehler [32] tocompute SPCA using an L1-based regularization to minimize the number of contributing items toward each principal component. The compromise between the cardinality (number of contributing items in each principal component) versus the explained variance was optimized using trial and error in order to obtain principal components that explained as much of the variance as possible, while still being easily interpretable.

### Density Plots and Statistical Hypothesis Testing

We computed the densities using kernel density estimation with Gaussian kernels to visualize the differences in the latent variables for the three cohorts and used the 2-sample Kolmogorov-Smirnov goodness-of-fit statistical hypothesis test to determine whether the distributions are statistically significantly different. We tested the null hypothesis that the random samples are drawn from the same underlying continuous distribution.

### Differentiating Cohorts Using Divergence Metrics

Next, we wanted to quantify the difference in the distributions of the principal components for the different groups. The computation of effect sizes is one widely used approach to quantify these differences, but relies on having Gaussian distributions which is not necessarily the case here. A more generic methodology to quantify differences between two distributions relies on the divergence metrics [33,34]. The divergence metrics make no strong hypotheses about the underlying distributions (primarily that they exist and are continuous) and can be thought of as robust approaches to measure how much two distributions differ. Here, we report the commonly used symmetric Kullback-Leibler divergence to quantify differences between two distributions. The distributions were computed using kernel density estimation with Gaussian kernels.

## Results

### Latent Variable Questionnaire Structure

Table 2 presents the latent variable structure for the MZ questionnaire using the standard PCA. The tentative labeling of the resulting principal components was driven by the members of the AMoSS team with clinical background and decided by consensus from all authors. The presence of non-zero loadings for all items obstructs the clear interpretation of the underlying meaning of the principal components. For example, the first principal component for BD and BPD could be tentatively interpreted as “negative affect” since the MZ items with a negative connotation tend to dominate; nevertheless, there is non-negligible contribution by all items thus complicating the task of understanding the latent variable meaning. Similarly, the second principal component could be considered to denote the “positive affect” since the 2 key items with large loadings denote positive feelings; nevertheless, there is some nonnegligible contribution from the remaining items. Moreover, it is not easy to interpret the third principal component (henceforth, when a latent variable cannot be interpreted in a simple term, it is left blank). These findings motivated the search for computing sparse principal components.

In Table 3 we present the findings using SPCA, which leads to more interpretable latent variables. We note that in this case the results are more intuitively understandable compared with Table 2, since the loading matrix comprises many non-contributing items toward the computation of the principal components. Crucially, the principal components are identical for the 3 groups (with different order), supporting the concept of a coherent internal MZ latent variable structure in the study of the 3 cohorts investigated here. Furthermore, the results reported using SPCA in Table 3 provide further intuitive understanding into the key latent variables of Table 2; essentially, the “negative affect” was decomposed into its two constituents, “anxiety and sadness” and “irritability,” while the “Positive affect” seen in Table 2 remained unaffected. Finally, the order of the principal components for each of the 3 cohorts is revealing about the latent variables which are most predictive in each case: for the patient cohorts, anxiety and sadness appears to be the most important mood symptom characteristic, whereas in HC most of the variance is explained using the “positive affect.”

Next, we applied SPCA on ASRM (Table 4), QIDS (Table 5), and GAD-7 (Table 6). The aim was to determine how stable the latent variables of each questionnaire are across groups, and determine whether there are some latent variables common across the investigated questionnaires and MZ.

The latent variable structure of ASRM is not consistent across the 3 groups, but it is consistent for the psychiatric groups. Some of the computed latent variables are not easily interpretable: for example, it is not clear how we should interpret the latent variable consisting of the items “sleepy” and “talkative.” The “positive affect” in the ASRM latent variable reported in Table 4 for BD and BPD appears to be very similar with the “positive affect” reported in Table 3 for MZ. This is a finding that could have been reasonably expected on the basis of the key items identified for the 2 questionnaires. In general, the HC participants in AMoSS did not exhibit manic episodes and their ASRM variability was very low. Thus, the findings for the HC group should be interpreted very cautiously as possibly due to lack of data.

**Table 2:** Mood zoom (MZ) latent variable structure using standard principal component analysis (PCA).

MZ item		P1	P2	P3
**Bipolar disorder**				
	Anxious	0.52	0.10	0.81
	Elated	−0.19	0.72	0.07
	Sad	0.49	0.09	−0.05
	Angry	0.45	0.17	−0.44
	Irritable	0.47	0.19	−0.38
	Energetic	−0.19	0.63	0.03
	% total variance explained	57.8	77.2	84.6
	Tentative interpretation	Negative affect	Positive affect	
**Borderline personality disorder**				
	Anxious	0.51	−0.01	0.39
	Elated	−0.13	0.70	0.24
	Sad	0.48	−0.24	0.56
	Angry	0.48	0.24	−0.36
	Irritable	0.51	0.27	−0.49
	Energetic	−0.07	0.58	0.32
	% total variance explained	48.9	69.6	81.2
	Tentative interpretation	Negative affect	Positive affect	
**Healthy controls**				
	Anxious	0.18	0.57	−0.06
	Elated	0.74	−0.23	−0.63
	Sad	0.15	0.50	−0.02
	Angry	0.12	0.37	0.03
	Irritable	0.12	0.46	0.05
	Energetic	0.61	−0.17	0.77
	% total variance explained	51.7	78.2	87.8
	Tentative interpretation	Positive affect	Negative affect	

**Table 3:** Sparse mood zoom (MZ) latent variable structure.

MZ item		P1	P2	P3
**Bipolar disorder**				
	Anxious	0.75	0	0
	Elated	0	−0.64	0
	Sad	0.66	0	0
	Angry	0	0	0.62
	Irritable	0	0	0.79
	Energetic	0	−0.77	0
	% total variance explained	33.1	56.6	75.8
	Tentative interpretation	Anxiety and sadness	Positive affect	Irritability
**Borderline personality disorder**				
	Anxious	0.66	0	0
	Elated	0	0	−0.71
	Sad	0.75	0	0
	Angry	0	0.67	0
	Irritable	0	0.74	0
	Energetic	0	0	−0.70
	% total variance explained	31.5	54.9	74.7
	Tentative interpretation	Anxiety and sadness	Irritability	Positive affect
**Healthy controls**				
	Anxious	0	0.73	0
	Elated	−0.66	0	0
	Sad	0	0.68	0
	Angry	0	0	−0.59
	Irritable	0	0	−0.81
	Energetic	−0.75	0	0
	% total variance explained	37.9	58.9	73.5
	Tentative interpretation	Positive affect	Anxiety and sadness	Irritability

QIDS appears to have a very inconsistent structure when examined with SPCA. In most cases, it is not easy to interpret what the resulting principal components mean; this may reflect that the QIDS items are disjoint, and there is no clear underlying latent variable structure.

GAD-7, like QIDS, is not very consistent across the 3 cohorts. Moreover, some of the resulting latent variables are difficult to interpret, for example, the meaning of the principal component comprised of the items “relaxed” and “restless.” Nevertheless, some of the latent variables across cohorts are consistent: the latent variable “nervousness” explains most of the variance in HC and BD. This is effectively the equivalent latent variable of MZ “anxiety and sadness” in Table 3.

**Table 4:** Sparse Altman self-rating mania (ASRM) scale latent variable structure.

ASRM item		P1	P2	P3
**Bipolar disorder**				
	Happy	0.65	0	−0.45
	Confident	0	0	−0.89
	Sleepy	0	0.92	0
	Talkative	0	0.38	0
	Active	0.76	0	0
	% total variance explained	50.1	69.1	82
	Tentative interpretation	Positive affect	Sleepy and talkative	Assertiveness
**Borderline personality disorder**				
	Happy	0.57	0	−0.58
	Confident	0	0	−0.81
	Sleepy	0	0.88	0
	Talkative	0	0.47	0
	Active	0.82	0	0
	% total variance explained	47.4	67	80.9
	Tentative interpretation	Positive affect	Sleepy and talkative	Assertiveness
**Healthy controls**				
	Happy	0.90	0	0
	Confident	0.44	0	0
	Sleepy	0	0	0
	Talkative	0	0.31	−0.95
	Active	0	0.95	0.31
	% total variance explained	39.7	66.2	79.9
	Tentative interpretation	Assertiveness	Active and talkative	Quiet and active

### Differentiating Cohorts

We investigated whether the principal components could differentiate the 3 cohorts in the study, BD, BPD, and HC. Since only MZ has a consistent latent variable structure across all 3 cohorts, the comparisons are only reported for that questionnaire in Table 7.

**Figure 1:**
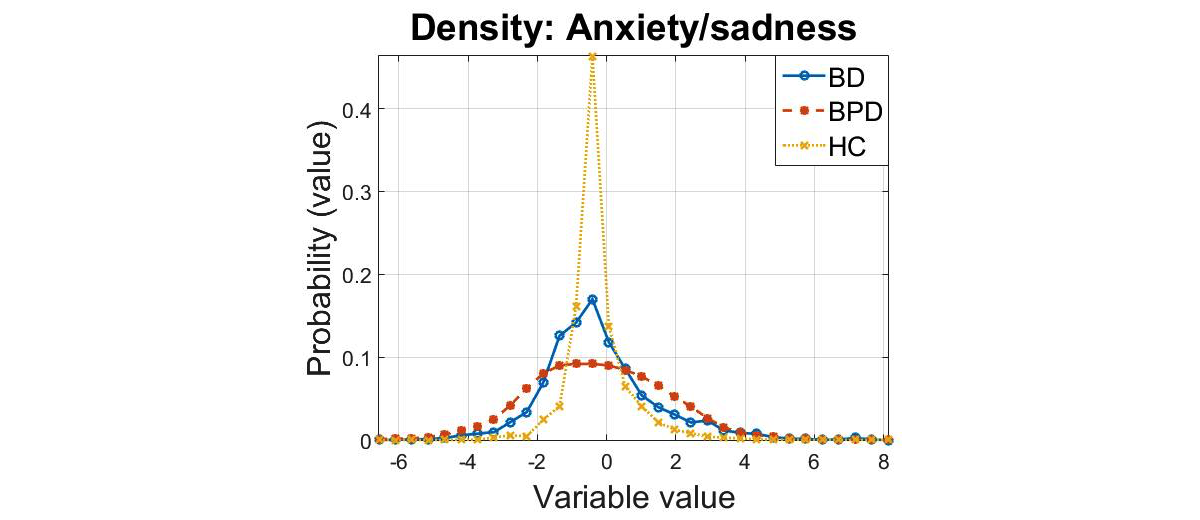
Density estimates of the “anxiety and sadness” principal component for the three cohorts.

**Figure 2:**
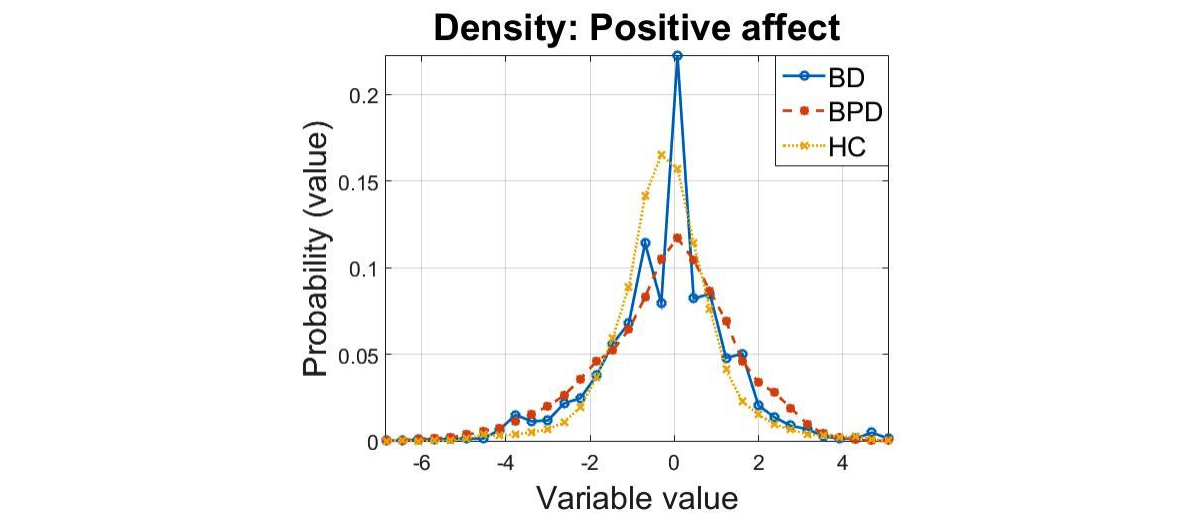
Density estimates of the “positive affect” principal component for the three cohorts.

**Figure 3:**
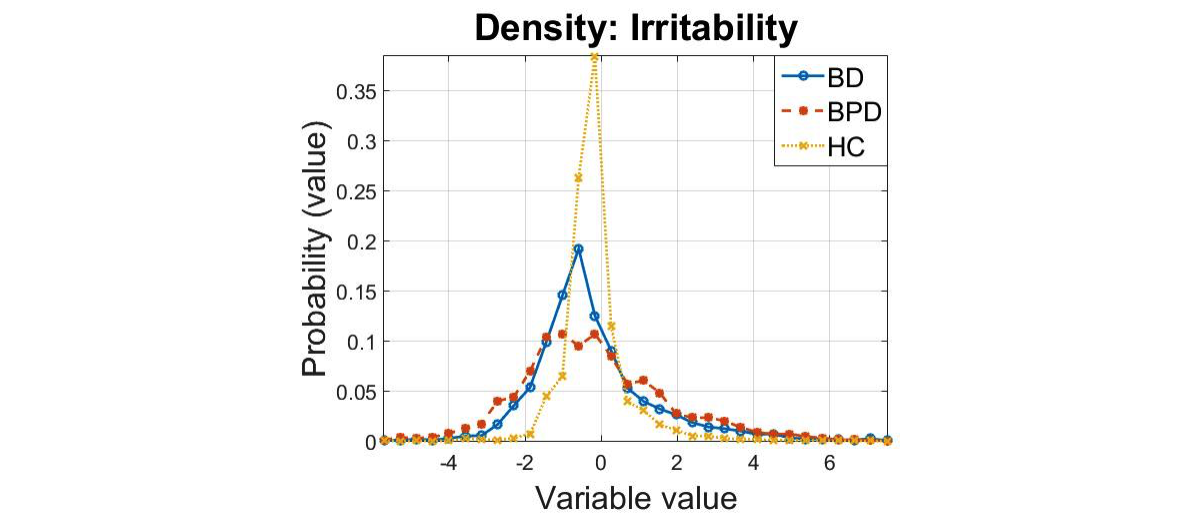
Density estimates of the “irritability” principal component for the three cohorts.

The densities of the principal components for the 3 cohorts are presented in Figures 1,2, and 3. In all cases, we found that the 2-sample Kolmogorov-Smirnov test rejected the null hypothesis that the samples were drawn from the same distribution, for all comparisons (*P*=0.001) this verifies the results expected following visual inspection of the densities.

We summarized the MZ latent variable values and quantified the differences between pairs of distributions using the symmetric Kullback-Leibler divergence in Table 7.

Overall, the findings in Table 7 suggest that the computed sparse principal components can adequately differentiate cohorts for all pairwise comparisons. We remark that the “irritability” principal component leads to clearer separation visually, a finding which is also reflected in the divergence values reported in Table 7. These results suggest that “irritability” swings may be one of the crucial differentiating factors between these 2 psychiatric cohorts.

**Table 5:** Sparse quick inventory of depressive symptomatology (QIDS) self-report latent variable structure.

QIDS item		P1		P2		P3	
**Bipolar disorder**								
	Sleep		0		−0.96		0
	Sad		−0.72		0		0
	Appetite or weight		0		0		−0.98
	Concentration		0		0		0
	Self-view		−0.69		0		0
	Suicide		0		0		0
	Interest		0		0		0
	Energy		0		0		−0.22
	Restless		0		-0.28		0
	% total variance explained		30.2		50.4		68.4
	Tentative interpretation		Esteem and sadness		Sleep changes		Appetite and energy
**Borderline personality disorder**								
	Sleep		0		0		0
	Sad		0		0		0
	Appetite or weight		0		−0.94		0
	Concentration		0		0		0
	Self-view		0		0		0.89
	Suicide		0		0		0.45
	Interest		−0.78		0		0
	Energy		−0.62		0		0
	Restless		0		−0.33		0
	% total variance explained		31.2		50.3		68
	Tentative interpretation		Energetic		Appetite and restlessness		Self-esteem and suicide
**Healthy controls**								
	Sleep		−0.99		0		0
	Sad		0		0		−0.83
	Appetite or weight		0		−0.96		0
	Concentration		0		0		0
	Self-view		0		0		−0.55
	Suicide		0		0		0
	Interest		0		0		0
	Energy		−0.15		−0.29		0
	Restless		0		0		0
	% total variance explained		37.9		59.7		76
	Tentative interpretation		Sleep		Appetite and energy		Esteem and sadness

**Table 6:** Sparse generalized anxiety disorder 7 (GAD-7) latent variable structure.

GAD-7 item			P1		P2		P3
**Bipolar disorder**							
	Nervous or anxious	−0.75		0		0	
	Control worries	−0.67		0		0	
	Worried	0		0		0	
	Relaxed	0		−0.37		0.54	
	Restless	0		0		0.84	
	Irritable	0		−0.93		0	
	Afraid	0		0		0	
	% total variance explained	41.2		60.5		72.9	
	Tentative interpretation	Nervousness		Irritability and relaxation		Activity	
**Borderline personality disorder**							
	Nervous or anxious	0		0		0	
	Control worries	0		0		−0.71	
	Worried	0		0		−0.70	
	Relaxed	0.63		0		0	
	Restless	0.78		0		0	
	Irritable	0		0.81		0	
	Afraid	0		0.58		0	
	% total variance explained	29.3		48.4		69.8	
	Tentative interpretation	Activity		Irritability and fear		Worry	
**Healthy controls**							
	Nervous or anxious	0.81		0		0	
	Control worries	0.58		0		0.46	
	Worried	0		−0.23		0.89	
	Relaxed	0		0		0	
	Restless	0		0		0	
	Irritable	0		−0.97		0	
	Afraid	0		0		0	
	% total variance explained	36.2		59.9		73.5	
	Tentative interpretation	Nervousness		Irritability and worry		Worry	

**Table 7:** Summary statistics for the sparse principal components computed in Table 3, and symmetric Kullback-Leibler divergence for pairwise comparisons across the 3 groups (BD, BPD, HC).

Sparse principal component		BD^a^ Median (IQR^d^)	BPD^b^ Median (IQR)	HC^c^ Median (IQR)	BD versus BPD (divergence)	BD versus HC (divergence)	BPD versus HC (divergence)
**Mood Zoom**							
	P1^e^	−0.16 (1.89)	−0.12 (2.56)	−0.08 (0.63)	1.78	4.46	4.72
	P2^f^	0.16 (1.60)	0.11 (1.98)	0.03 (1.29)	1.15	0.97	1.25
	P3^g^	−0.27 (1.47)	−0.16 (2.31)	−0.05 (0.34)	3.67	3.17	6.78

^a^BD: bipolar disorder.

^b^BPD: borderline personality disorder.

^c^HC: healthy controls.

^d^IQR: interquartile range.

^e^P1= “anxiety and sadness.”

^f^P2= “positive affect.”

^g^P3= “irritability.”

## Discussion

### Principal Findings

We have applied a recently developed form of SPCA to explore the latent variables of four psychiatric questionnaires across BD, BPD, and HC. We emphasize that the SPCA used here was guided primarily by the need to develop simple latent variables that would facilitate interpretation over and above findings computed using the standard PCA. As expected, in most cases the loadings in the patient cohorts were more similar compared with HC. The latent variable structure was stable across all three cohorts for MZ and stable across the patient cohorts for ASRM. On the contrary, the latent variable structure was quite different for the three cohorts for QIDS and GAD-7. Broadly speaking, having the same latent variables across cohorts indicates internal consistency of a questionnaire and is a convenient property because it enables direct quantitative comparisons of the resulting latent variables (see Table 7). On the other hand, having different resulting latent variables across cohorts could lead to the identification of the most prominent mood item cluster constellations in each case.

The recently proposed MZ [9] can be described in terms of three latent variables which can be tentatively interpreted as (1) anxiety and sadness, (2) irritability, and (3) positive affect. These three latent variables explain about 75% of the variance (Table 3), which is consistent across the three studied cohorts (BD, BPD, and HC). Moreover, the anxiety and sadness principal component explains most of the variance for the BD and BPD cohorts, while the positive affect explains most of the variance for HC. Similarly, the primary latent variable in GAD-7 for BD was “nervousness.” Thus, BD participants are strongly affected by anxiety, which is known to be a common comorbid factor [35]; this further supports the argument that anxiety should be customarily monitored longitudinally in addition to the cornerstone mania and depression symptoms [17]. However, the first two MZ latent variables appear to have considerable overlap between the psychiatric groups. The latent variable that differentiates BD from BPD best is “irritability” (see Table 7). Our findings suggest that BPD participants exhibit considerably larger irritability variability compared with BD participants. Further work is required to investigate how this finding might be used by psychiatrists in the challenging setting of differential diagnosis between the 2 groups [36].

The latent variable structure of ASRM was identical for BD and BPD but differed when compared with HC; this may indicate that the psychiatric groups have the same underlying effects when reporting mania symptoms. However, we view this finding very cautiously, because the ASRM variability was extremely low for HC. Sleep appears to be a key item in the latent variables of QIDS for HC and BD but not BPD. This might reflect a true difference in the perception of the effect of sleep on mood symptoms in BPD; again, this finding should be treated with caution because most BPD participants in the study were unemployed and hence, this may have skewed their responses.

It is difficult to cross-reference the questionnaires since they have been fundamentally developed to capture different mood symptoms (ASRM for mania, QIDS for depression, and GAD-7 for anxiety). Nevertheless, we have seen that irritability is a key latent variable in MZ, and that item dominates the second latent variable in GAD-7. Similarly, “anxiety and sadness” is the primary latent variable of MZ, which is similar to the first latent variable observed for BD and HC in GAD-7 (Table 6). To test whether we can obtain cross-referenced latent variables among questionnaires, we merged ASRM, QIDS, and GAD-7 in a single dataset and applied SPCA for each of the three cohorts (results not shown). In almost all cases, the latent variables computed were clustered within the items of the same questionnaire and were typically dominated by QIDS items, with findings similar to those reported in Table 6. This suggests that depression-related symptoms explain most of the variance overall across the three questionnaires, a finding which is in agreement with the BD literature [15].

Understanding and interpreting the latent variables may have important implications for understanding mood traits and mood trait interactions and could lead into new hypotheses and clinical research insights. We found that anxiety and sadness are mood characteristics that covary consistently across groups (Table 3) indicating they are comorbid symptoms [37], and corroborating contemporary clinical practice treatment approaches often jointly addressing both [38]. Similarly, the latent variable comprising the items “elated” and “energetic” (Table 3) suggests there is a general underlying feeling of positive affect linking euphoria and energy. Crucially, this latent variable was found to be explaining most of the variance in the data for HC but not for the patient groups. The last MZ latent variable comprises the “angry” and “irritable” items, in line with results reported by Pasquini et al [39] who studied major depressive disorder using a very different clinical scale and processed their data using factor analysis to derive the same component. The current study’s results generalize their main conclusion that psychiatrists should be aware of the relevance of this dimension in assessment and treatment of BD and BPD. The latent variable which we called “assertiveness” (Table 4) indicates that the “happy” and “confident” items cluster together across all three cohorts and is particularly prominent in HC explaining most of the variance. This finding may have wider implications suggesting that increasing someone’s perceived happiness may also boost confidence. We also reported on a latent variable comprising sadness and low self-esteem (Table 5), which is common in BD and HC; some studies have empirically linked depleted self-esteem with increased depressive symptoms [40]. The corresponding latent variable for BPD comprises the intricately intertwined “self-esteem” and “suicide” items; hence, low self-esteem may have considerably more severe consequences for this patient group compared with BD, suggesting experts may need to be particularly vigilant in the morale of their BPD patients. Finally, in Table 6 the irritability item dominates the second latent variable of GAD-7 in all cohorts; however, it is grouped with a different item in each case: (1) “relaxation” for BD, (2) “fear” for BPD, and (3) “worry” for HC. Hence, the mood trait expressed in the “angry” item in MZ appears to act as an umbrella term capturing different mood aspects that appear in GAD-7 for each of the three cohorts.

### Comparison With Prior Work

We have presented results from a relatively large number of participants in the context of longitudinal mood monitoring, tracking their mood variation for multiple months as opposed to other studies, which were confined to a few weeks (eg, [7,41]). Moreover, we elicited answers to multiple questionnaires, whereas most studies had focused on a single questionnaire to investigate symptom variation, for example, depression [41-43]. Additionally, most other studies focus solely on a single disorder, for example, BD [5,41-43], whereas we have also recruited people diagnosed with BPD and compared findings against HC.

There is a large number of PROMs developed for (1) the general population, (2) broad population cohorts (eg, people diagnosed with mental disorders), and (3) specific disorders such as BD. Well-known generic instruments include the profile of moods state (POMS) [44] and the positive and negative affectivity schedule (PANAS) [45]. The full-length form of POMS comprises 65 items whereas the short form comprises 35 items [44]; the user would likely need 5-10 min to complete these. Based on the original items, POMS computes the participant’s mood profile comprising the following mood dimensions: (1) anger-hostility, (2) confusion-bewilderment, (3) depression-dejection, (4) fatigue-inertia, (5) tensor-anxiety, (6) vigor-activity, and (7) friendliness. Although these seven dimensions bear similarities with the 6 MZ items, we emphasize that the two methods actually exhibit some differences in terms of the mood profiles assessed, and more importantly have very different approaches at how these mood characteristics are computed. They are evaluated directly on a 7-point Likert scale in MZ, whereas they are computed in POMS from the originally 35 or 65 items, each of which is rated on a 5-point Likert scale. PANAS comprises 20 items in total (10 for positive affect, 10 for negative affect), each of which is rated on a 5-point Likert scale. Again, although there is some overlap in terms of the items used in PANAS and MZ, the two methods are different both in terms of the actual items used (for example PANAS does not include the MZ items “anxious” and “sad”) and also in terms of the Likert scale length (5-point for PANAS). Therefore, MZ has subtle but important differences when compared with POMS and PANAS. The major advantage of MZ is that it is a very compact questionnaire developed primarily to capture the main mood swings in BD and BPD, while at the same time fitting a smartphone screen [9]. Thus, its completion takes only a couple of seconds, which is likely a critical aspect when requesting participants to fill in a questionnaire daily and longitudinally, and it is probably one of the reasons it was well-received and led to over 80% long-term adherence [9].

Alternative specialized PROM instruments such as the young mania rating scale (YMRS) [46] to assess mania symptoms and patient health questionnaire-9 (PHQ-9) [47] to assess depressive symptoms have been used in some related studies. It is difficult to argue which measure is more appropriate in either case. The use of ASRM and QIDS in this study over YMRS and PHQ-9 reflects more a pragmatic legacy approach; many of the BD participants in the AMoSS study have been recruited from a larger study where they have been reporting ASRM and QIDS for several years (in some cases more than 7 years) as part of the Oxford NHS TC system. Therefore, at the beginning of the study, we decided to continue using these questionnaires that will enable long-term BD monitoring on the same clinical scales and might provide further insight into seasonality effects and long-term symptom changes.

Clinical diagnosis of mental disorders has traditionally relied on conventional DSM guidelines, which is a symptom-based approach. A relatively recently proposed framework for studying mental disorders is the research domain criteria (RDoC), which aims to provide a more inclusive, multidimensional approach including genetic, neural, and behavioral features [48]. One of the RDoC dimensions is “self-reports” (interview scales, questionnaires) and is assessed on items comprising the latent categories “negative valence” (anxiety, fear) and “positive valence” (motivation, responsiveness). Therefore, there is some overlap in the computed MZ latent variables and the suggested RDoC self-reports dimension. We remark that the RDoC was conceived as a diagnostic category agnostic framework to be adapted by researchers based on their needs, proposing a continuum of assessment rather than a categorical-based assessment. This study’s findings could be used to inform the self-reports dimension of the RDoC, particularly since we found the MZ latent variables to be stable across the psychiatric cohorts and HC.

Although some previous studies have studied the internal consistency of psychiatric questionnaires [49,50], to the best of our knowledge, no study has investigated the internal structure of different questionnaires across psychiatric groups using SPCA to obtain interpretable latent variables. One method that has often been successfully used to compute latent variables in the field of psychiatry is item response theory (IRT), for example, see Rush et al [23]. Mathematically, IRT operates on discrete data; however, since we process datasets comprising attributes in the continuous domain, the implicit assumptions for using IRT are not valid.

In a recent previous study [9], we had introduced MZ and used PCA to investigate its properties. We had reported two principal components across the three cohorts (BD, BPD, and HC), which we referred to as “negative MZ” and “positive MZ.” The presence of a positive affect and negative affect had been previously described in studies of normal emotion in psychology [51]. This study’s findings provide further insight into the “negative affect” MZ; it can be further decomposed into two components, which we interpret as “anxiety and sadness” and “irritability.”

### Limitations

Notwithstanding the relatively large number of participants for the studied patient groups, there were certain limitations. First, we used three widely established questionnaires used for self-assessment of mood symptoms (ASRM, QIDS, and GAD-7) and the recently proposed MZ. There are numerous other questionnaires in the psychiatric literature, some of which have also been used in the context of BD.

Second, most of the BD participants were recruited from a larger study; therefore, they might be more compliant than a new cohort in this diagnostic group. However, we stress that participants were originally recruited for 3 months with the option to stay longer; the majority found the study engaging and provided data for at least a year. Although the study cohort was representative of a subgroup of psychiatric outpatients, it did not include those who were psychotic or who had significant comorbidities. Moreover, the vast majority of the BD cohort was euthymic for the larger part of the AMoSS study with very few participants exhibiting the characteristic alternating periods of mania and depression. Future studies could investigate differences within BD to compare questionnaire latent variable structures and loadings of a euthymic subgroup versus a subgroup cycling through mania and depression.

Third, the study was observational in nature, and we had very little contact with participants. The pharmacological treatment at trial onset was recorded, but we do not have accurate information on changes in medication through the duration of the study. All the reported scores rely on self-assessment; there is a lack of ongoing clinical assessment by experts to validate the findings. For example, Faurholt-Jepsen et al [52], in a meta-analysis study, reported that self-reported measures on mania may not be reflective of the true clinical condition.

Finally, there are multiple machine learning techniques to determine the latent variable structure of the data. In addition to different types of SPCA with different penalties and regularization settings, there are alternative techniques such as factor analysis, non-negative matrix factorization, and more complicated manifold embedding methods [28,53]. Ultimately, all these algorithms need to balance between the explanatory power and the interpretability of the computed latent variables. Future studies could investigate further into more complicated schemes and latent variable structures.

We tried to identify the underlying psychological processes for the three cohorts by interpreting the latent variables computed from a single modality: self-assessed questionnaires. It could be argued that using latent variables compared with single items might be more robust in defining underlying psychological processes because they rely on multiple items which covary, and hence these provide a better means to identify differences between cohorts. Nevertheless, this argument would need to be validated using additional data looking at more detailed aspects about how these facets overlap with markers from other modalities. We have collected a large set of additional modalities in AMoSS (electrocardiogram, geolocation, activity, sleep, and social interaction) which we will be exploring in future work. Ultimately, as suggested in RDoC, mental health is not a single-dimensional concept, and fusing information from multiple modalities can bring additional key insights and improve understanding of the underlying processes and clinical assessment.

### Conclusions

The findings in this study further support the recent introduction of MZ in clinical psychiatric practice. Its structure in terms of the first three principal components is consistent across BD, BPD, and HC, and the order of the principal components can be tentatively understood intuitively. ASRM is consistent for the patient groups versus HC. QIDS and GAD-7 are more varied and do not lead to easily interpretable principal components. We found that BD and BPD are very similar in terms of some standardized questionnaires (ASRM) but quite divergent in terms of QIDS and GAD-7. Further work is warranted to understand the similarities and differences between BD and BPD, which may facilitate differential diagnosis and long-term monitoring of their treatment approaches.

## Abbreviations

AMoSS: automated monitoring of symptom severity
ASRM: Altman self-rating mania
BD: bipolar disorder
BPD: borderline personality disorder
DSM: diagnostic and statistical manual of mental disorders
GAD-7: generalized anxiety disorder (7-item)
QIDS: quick inventory of depressive symptomatology
HC: healthy controls
IPDE: international personality disorder examination
MZ: mood zoom
PANAS: positive and negative affectivity schedule
PCA: principal component analysis
POMS: profile of moods state
PROM: patient reported outcome measures
RDoC: research domain criteria
SPCA: sparse principal component analysis
TC: true colors
YMRS: young mania rating scale
